# Upscaling the Hyperpolarization Sample Volume of an Automated Hydrogenative Parahydrogen-Induced Polarizer

**DOI:** 10.1021/acsmeasuresciau.5c00097

**Published:** 2025-10-06

**Authors:** Yenal Gökpek, Jan-Bernd Hövener, Andrey N. Pravdivtsev

**Affiliations:** Section Biomedical Imaging, Molecular Imaging North Competence Center (MOIN CC), Department of Radiology and Neuroradiology, University Hospital Schleswig-Holstein, Kiel University, Am Botanischen Garten 14, 24114 Kiel, Germany

**Keywords:** PHIP-SAH, hyperpolarization, vinyl acetate, automation, parahydrogen

## Abstract

Nuclear magnetic resonance (NMR) and magnetic resonance imaging (MRI) suffer from inherently low sensitivity due to the weak thermal polarization of nuclear spins. Parahydrogen-induced polarization (PHIP) offers a powerful route to enhance NMR signals by several orders of magnitude, enabling real-time metabolic imaging. However, PHIP implementations are often constrained by small sample volumes, limited automation, and complex high-pressure requirements. In this work, we present an upgraded, automated PHIP system capable of hyperpolarizing sample volumes up to 2.2 mL, which is suitable for preclinical MRI applications. We developed several high-pressure reactors and multiport NMR tube caps compatible with standard commercial 5, 10, and 16 mm glass tubes. Reactor designs were simulated and fabricated from chemically resistant polymers, ensuring mechanical safety at more than 30 bar. Using FLASH MRI, nutation, and CPMG sequences, we characterized magnetic field homogeneity and stability, establishing optimal sample dimensions (12.5/16 mm ID/OD glass tube, 20 mm height) with a *B*_0_ inhomogeneity below 2.5 ppm and a *B*_1_ inhomogeneity around 1%. A high level of injection reproducibility was confirmed (volume precision ∼ 0.6%). Optimization of experimental parameters, including the hydrogenation pressure, pH_2_ flow rate, and sample temperature, enabled rapid and efficient polarization transfer. At optimized conditions (20 bar pH_2_, 2 L/min flow, 55 °C, 4 s bubbling time), up to 31.3% ^1^H polarization of two protons was achieved for deuterated ethyl acetate in acetone with the theoretical maximum of 50%. This level of polarization was achieved with a duty cycle of 80 s, and the coefficient of variation of the mean was below 6.8%. This system lays the groundwork for the broader adoption of PHIP in preclinical imaging and metabolic research, providing practical sample volumes and facilitating the rapid production of hyperpolarization. Future work includes automating the purification process and further maximization of the polarization yield.

## Graphical abstract

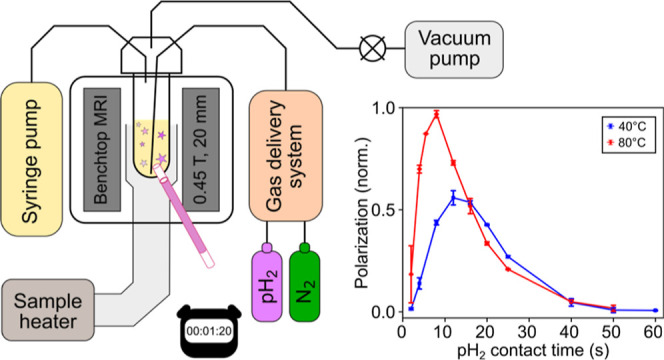

## Introduction

Nuclear magnetic resonance (NMR) uses weak nuclear spin interactions with the magnetic field to gain insights into the molecular structure and sample composition.^1^ Combined with magnetic field gradients, it enables noninvasive magnetic resonance imaging (MRI).^2^ However, the sensitivity of NMR is low, partially because of the low nuclear spin polarization: at fields of 9.4 T and 300 K, the polarization of ^1^H is about 3.2 × 10^–5^. Thus, even at 9.4 T, the MR signal can be enhanced up to 31.000 times when unity polarization is reached. Consequently, much effort is spent on researching methods to increase polarization.^3,4^ Dissolution dynamic nuclear polarization (DNP),^5,6^ parahydrogen-induced nuclear polarization (PHIP),^4,7–9^ and signal amplification by reversible exchange (SABRE)^10^ are among the most popular methods for hyperpolarizing molecules in solution. Remarkably, hyperpolarized MRI has enabled real-time noninvasive observation of metabolic transformations in vitro^11–13^ and in vivo.^14,15^

Hydrogenative PHIP typically utilizes a precursor with an unsaturated double or triple C–C bond, parahydrogen (pH_2_), and a catalyst to promote the hydrogenative reaction.^16–18^ pH_2_ is a lower-energy, singlet nuclear spin isomer of dihydrogen with a total nuclear spin of 0. It can be conveniently produced by cooling H_2_ gas to 77 K, where pH_2_ is enriched to ∼50% ^19,20^ or 25 K and below, where the enrichment is close to 100%.^21,22^

To polarize biomolecules like pyruvate and acetate, which do not have a native unsaturated precursor, an unsaturated side arm can be added to receive pH_2_. After hydrogenation, the polarization can be transferred to the target, and the side arm can be cleaved (Figure 1). This side arm hydrogenation (SAH) method (PHIP-SAH) was first proposed by Reineri et al.^23,24^ Its theory and practices were recently reviewed by Salnikov et al.^25^

**Figure 1:**
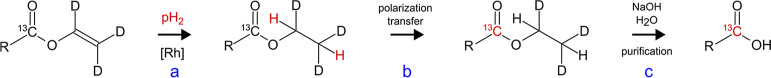
Schematic of the PHIP-SAH experiment. First, ^13^C-labeled deuterated vinyl ester with a residue group noted by R (acetate for R = CD_3_, pyruvate for R = COCD_3_) is hydrogenated to ethyl ester (a). Then, the pH_2_ spin order is converted into ^13^C spin magnetization (b). Finally, after the addition of NaOH to the aqueous solution to promote the cleavage of the side arm and purification, the ^13^C hyperpolarized ester was extracted (c). In this work, we used a vinyl acetate (VA) precursor, which, upon hydrogenation, yielded ethyl acetate (EA). Protonated (VA-*h*_6_) and deuterated (VA-*d*_6_) precursors were compared.

Most of the PHIP-SAH studies in the literature were conducted with 5 mm NMR tubes and small volumes (0.1–0.2 mL),^26–28^ which is reasonable in terms of resources. However, there are examples when larger-volume reaction vessels are constructed,^29,30^ or 10 mm NMR tubes are used.^31,32^

In general, PHIP experiments are rather complex and require well-defined and synchronized events of chemistry, fluidics, and NMR. Thus, since the beginning, a key element for well-defined and reproducible hyperpolarization experiments has been automation and quality control.^33–36^ For example, Schmidt et al.^37^ demonstrated a semiautomated approach in which a 0.7 mL sample was injected, infused with pH_2_, and ejected without purification every 15 s.

A commercial polarizer prototype for PHIP-SAH was also used by Nagel et al. for the preparation of nuclear spin hyperpolarization and consequent in vivo imaging.^38^

Recently, we introduced a semiautomated, PHIP-SAH compatible polarizer with a duty cycle of about 1 min, based on a permanent magnet portable MRI unit.^39^ The system operated with 10 mm NMR tubes at pH_2_ pressures of up to 30 bar. Larger samples suitable for animal imaging, however, were not feasible. Thus, we set out to develop a setup suitable for larger sample sizes in preclinical imaging.

Here, we present a novel polarizer and its performance for polarizing samples up to 2 mL, suitable for small animal imaging.^6,38,40^

First, we aimed to maximize the sample size (more than 2 mL) by varying the sample tube diameter (Figure 2) and sample height (Figure 3) while keeping the homogeneity of *B*_0_ (less than 5 ppm) and *B*_1_ across the sample. To achieve this goal, we developed and tested several PHIP reaction chambers based on high-pressure multiport NMR tube caps, 5 and 10 mm NMR tubes, and 16 mm microwave tubes. High *B*_0_ and *B*_1_ homogeneity throughout the entire sample is needed for a robust spin order transfer (SOT).^31^ On the other hand, automation enabled us to perform precise serial experiments with a duty cycle of 80 s, for example, by measuring the hydrogenation kinetics of vinyl acetate at various temperatures. After several iterations, we achieved a maximum average ^1^H polarization of 31.3% for two protons, out of a possible 44.6% for the used pH_2_ enrichment level. We believe that this is an essential step toward maximizing the hyperpolarization yield of automated hydrogenative PHIP.

**Figure 2:**
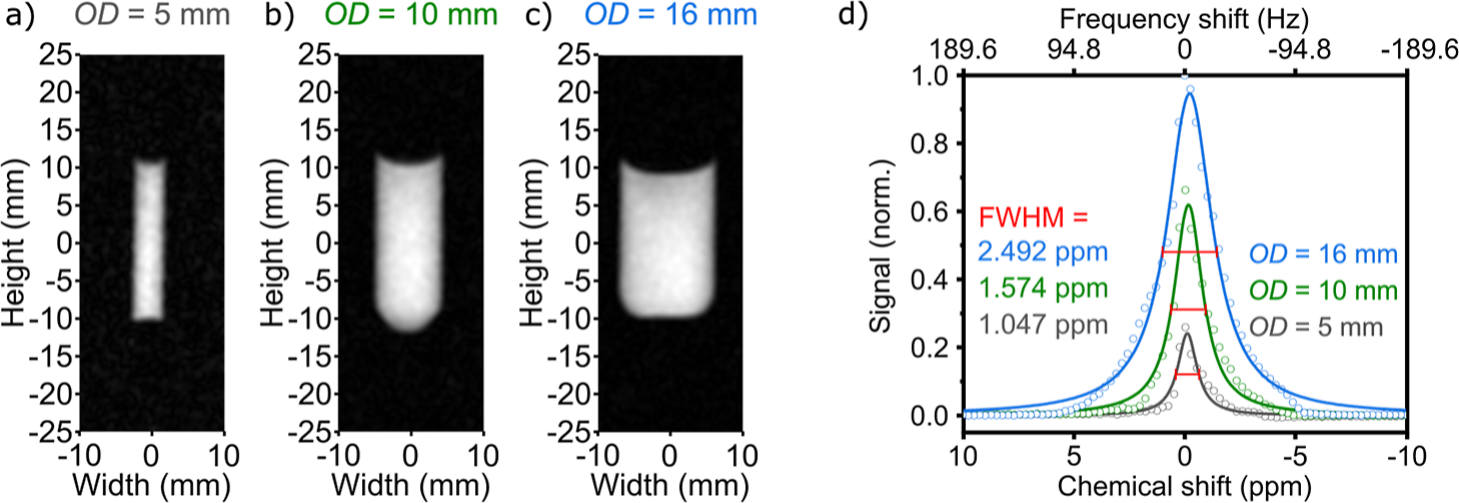
Effect of the sample’s diameter on the *B*_0_ homogeneity. ^1^H FLASH MRI of 5 mm (a), 10 mm (b), and 16 mm (c) OD sample tubes filled with 20 mm of acetone and corresponding nonlocalized ^1^H NMR spectra (d). The Lorentzian function fits (lines in part d) yielded FWHM values of 1.047, 1.574, and 2.492 ppm for 5, 10, and 16 mm sample tubes, respectively, under a *B*_0_ magnetic field of 0.4454 T. The (0,0) coordinate corresponds to the isocenter of the magnet.

**Figure 3:**
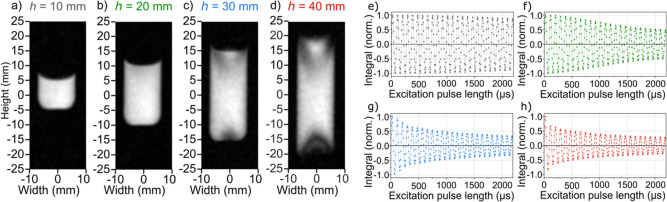
Effect of the sample height on *B*_0_ and *B*_1_ homogeneity. ^1^H FLASH MRI (a–d) and nutation curves (e–h) of acetone in the 16 mm OD reactor filled to heights, *h*, 10 (a,e), 20 (b,f), 30 (c,g), and 40 mm (d,h). A damped wave function *Ae* ^–*kt*^ sin­(2π*t*_pulse_/*T*) was fitted (continuous line) to the data and yielded *T* = (90.30 ± 0.14) μs and *k* = 57.8, 369.7, 521.8, and 531.9 s^–1^. *B*_0_ and *B*_1_ fields for samples up to *h* of 20 mm and OD of 16 mm are sufficiently homogeneous: in the case of *h* = 20 mm, the signal decreased by 6.7% after 5 periods.

## Results

Below, we describe how we characterized the conditions under which PHIP was performed, including the *B*_0_ and *B*_1_ fields of the MRI unit and the sample temperature. We further detail the design of PHIP reaction vessels, the optimization of hydrogenation parameters, and the application of an out-of-phase sequence for hyperpolarizing ethyl acetate (EA) in an inhomogeneous magnetic field, which enabled us to estimate the achieved ^1^H polarization.

### *B*_0_ Field Homogeneity: Sample Diameter

The *B*_0_ homogeneity was evaluated in 5 and 10 mm NMR tubes and a 16 mm OD microwave tube filled to different amounts (Figure 2, inner diameters (ID) 4.2 mm, 9.1 mm, and 12.5 mm). For 20 mm filling, nonlocalized ^1^H NMR of acetone yielded full widths at half-maximum (FWHM) of 1.047, 1.574, and 2.492 ppm (19.855, 29.847, and 47.254 Hz), for 5, 10, and 16 mm tubes, respectively.

In situ ^1^H FLASH MRI^2^ (Figure 2a–c) showed homogeneous images of the samples, indicating a sufficiently homogeneous *B*_0_ magnetic field across the sample. Thus, we continued to investigate the 16 mm reactor, which provided the desired volumes.

### *B*_0_ and *B*_1_ Field Homogeneity: Sample Height

We filled the 16 mm reactor to heights, *h*, of 10, 20, 30, and 40 mm and measured ^1^H spectra, ^1^H FLASH, and nutation curves (Figure 3, note that the FLASH images represent a combination of *B*_0_ and *B*_1_, while nutation curves assess only *B*_1_ homogeneity). The images were homogeneous for up to 20 mm filling and showed artifacts for *h* > 20 mm.

Fitting of nutation curves with a damped sine wave yielded decay rates of 57.8 ± 3.4, 369.7 ± 23.4, 521.8 ± 61.4, and 531.9 ± 76.6 s^–1^ for samples with 10, 20, 30, and 40 mm heights, respectively. Thus, the *B*_1_ homogeneities of the *h* = 30 and 40 mm samples were very similar. The nutation period was measured to be 90.30 ± 0.14 μs at a height of 20 mm. It could be seen that *B*_1_ homogeneity decreased with increasing sample height; however, the signal decay for the *h* = 20 mm sample after 5 nutation periods was only 6.7%.

To estimate the *B*_1_ field homogeneity more precisely, we simulated nutation curves as a decaying sine function, assuming a Gaussian distribution of the *B*_1_ field across the sample, and tried to estimate the *B*_1_ field homogeneity from this simulation in terms of standard deviation. We obtained the same nutation decay parameters as those experimentally obtained (Supporting Information, Figure S1). The resulting simulations yielded the following estimates for the relative standard deviations of the *B*_1_ fields: approximately 0.26%, 1.03%, 2.30%, and 4.08% for *h* = 10, 20, 30, and 40 mm, respectively. Hence, we concluded that the 20 mm bore magnet creates a sufficiently homogeneous magnetic field in the center of the field of view (FOV) with a 20 mm height and 12.5 mm width. These measurements indicated that the system is suitable for reactors with a sample size of (2.215 ± 0.014) mL, providing *B*_1_ inhomogeneity of about 1% and *B*_0_ inhomogeneity in ^1^H frequencies better than 47 Hz (2.492 ppm).

### *B*_0_ Field Stability: Measurement of *T*_2_ Relaxation Time

The *B*_1_ and *B*_0_ stability and homogeneity inside the 5, 10, and 16 mm reactors were measured with a Carr–Purcell–Meiboom–Gill (CPMG)^41,42^ sequence (Figure 4). The CPMG experiments were repeated using different echo time intervals, 2τ, from 2 to 800 ms, where 2τ is the time between two consecutive refocusing pulses. To ameliorate the effects of *B*_1_ inhomogeneity, we used composite pulses, 90_Y_^o^ 180_Y_^o^ 90_Y_^o^, instead of a single inversion pulse.^31^

**Figure 4:**
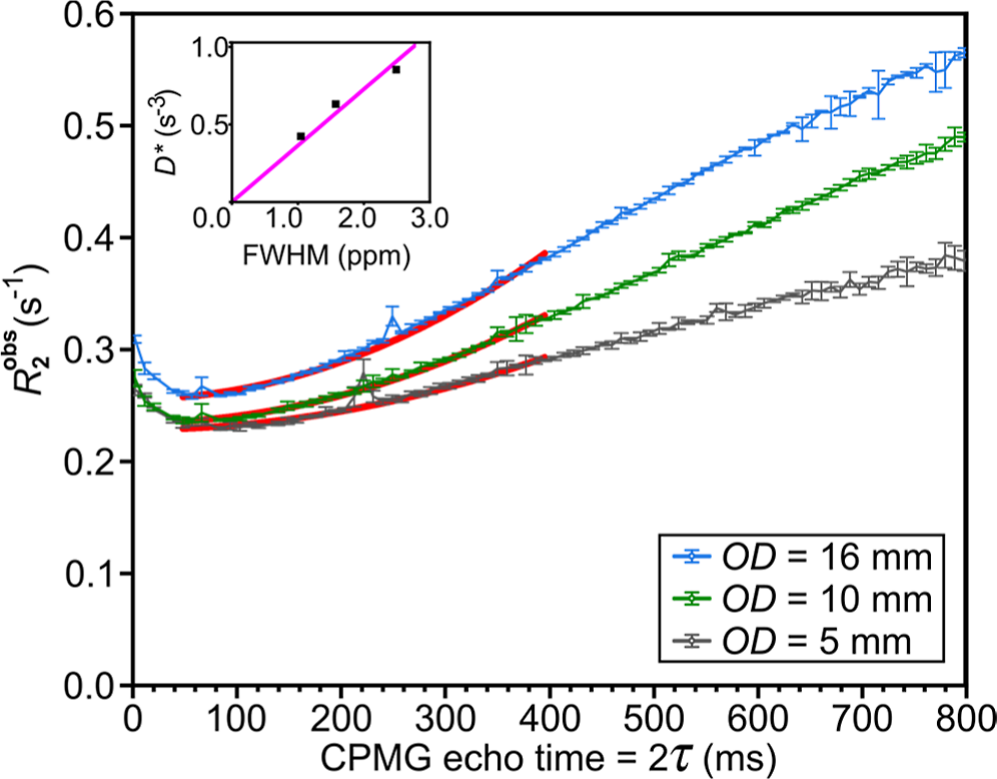
*T*_2_ relaxation of acetone. Observed *T*_2_ relaxation rate *R*_2_^obs^ as a function of CPMG echo time, 2τ, for 5, 10, and 16 mm OD tubes filled with acetone to a *h* of 20 mm. 169 echoes were measured for each τ value. *R*_2_^obs^ is a result of a monoexponential decay fitting, $M_{T}=M_{0}e^{-R_{2}^{obs}2n\tau }$ to the obtained signal decay for each τ. For 40 ms < 2τ < 400 ms, the Hahn equation, $R_{2}^{obs}=\frac{1}{T_{2}}+D^{*}{\left(2\tau \right)}^{2}$ , was fitted, yielding an effective diffusion-homogeneity coefficient D*. The inset demonstrates the correlation between D* and FWHM of the samples (pink line, linear fit). Whiskers indicate the standard deviation of 5 repeated experimental measurements.

The Hahn equation,^41^ $M_{T}=M_{0}e^{-R_{2}^{obs}2n\tau }$ , where is the transverse magnetization, *M*_0_ is the initial magnetization, *R*_2_^obs^ is the observed *T*_2_ relaxation decay rate, and *n* is the number of refocusing pulses, was used to fit the signal decay kinetics as a function of *n* for each τ.

In a perfectly stable and homogeneous static magnetic field, the signal decay should not vary with echo interval for acetone; in our case, however, it did change. The *R*_2_^obs^ values were fitted (Figure 4—red curves) between 40 and 400 ms of 2τ range with the equation accounting for *B*_0_ inhomogeneity and diffusion^39^ $R_{2}^{obs}=\frac{1}{T_{2}}+D^{*}{\left(2\tau \right)}^{2}$ , where D* is the effective diffusion-homogeneity coefficient, which is given by $D^{*}=\frac{1}{12}\gamma^{2}G^{2}D$ , where γ is the gyromagnetic ratio, *G* is the magnetic field gradient of the system caused by *B*_0_ inhomogeneity, and *D* is the ideal diffusion coefficient. The linear fit showed a high correlation of D* with the previously assessed FWHM (Figure 2d). Including the origin point (0,0) as a point to pass through, we obtained *R* ^2^ = 0.99895 (Figure 4—inset). This supported the hypothesis of the observed *R*_2_^obs^ dependency.

Here, we observed an unexpected increase in the *R*_2_^obs^ at lower τ values. This effect may result from enhanced sample convection caused by RF-induced heating, as well as from limitations in data fitting, since the 169 acquired echoes only permitted kinetic measurements over times shorter than one *T*_2_; therefore, values under 40 ms of 2τ were omitted from the fitting. Note that mispositioning of the sample can lead to inhomogeneous *B*_1_ and *B*_0_ fields, which in turn may produce erroneous *T*_2_ dependencies (Figure S8, Supporting Information).

For all ODs and sample heights of 20 mm, echo times below 50 ms appeared stable enough, providing similar observed *T*_2_; hence, for polarization transfer, the echo times should not exceed this value. The characterizing properties of the large bore NMR system, such as FWHM, *T*_2_^*^, *T*_2_, and D* with different OD tubes, are summarized in Table 1.

**Table 1:** FWHM, *T*_2_^*^, *T*_2_, and D* Values Measured for 5, 10, and 16 mm OD Tubes Filled with Acetone to a *h* of 20 mm^a^

reactor diameter (mm)	FWHM (Hz)	FWHM (ppm)	*T*_2_^*^ (s)	*T*_2_ (τ = 0)(s)	D* (s^–3^)
5	1.05	19.9	0.30	4.38 ± 0.01	0.41 ± 0.01
10	1.57	29.9	0.20	4.27 ± 0.01	0.61 ± 0.01
16	2.50	47.3	0.13	3.91 ± 0.01	0.83 ± 0.01

a Measurements were done with acetone at 40 °C and *B*_0_ = 0.4454 T. *T*_2_^*^ is estimated from the FWHM as 1/(π·FWHM). *T*_2_(*τ* = 0) and D* were obtained from the CPMG measurements.

### Reactor Design and Simulation

We designed 10 different reactors with variable properties to accommodate various experimental conditions and setups (Figure 5). The reactors were designed to increase the attainable pressure of pH_2_, enhance the hyperpolarization yield by accelerating the hydrogenation rate, increase volume, and reduce the costs associated with previously used, expensive proprietary high-pressure NMR tubes.

**Figure 5:**
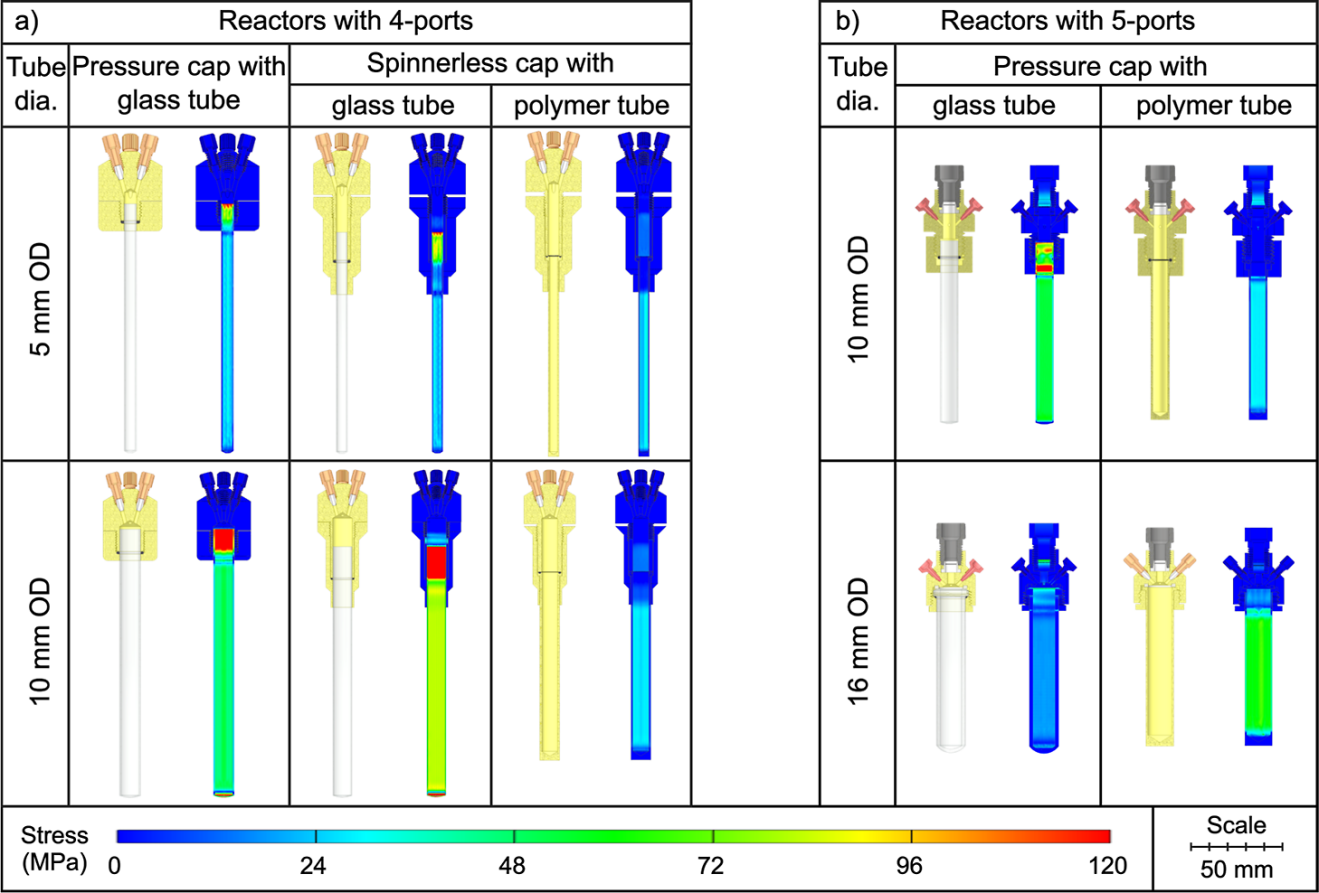
Overview of the designed pH_2_ reactors and pressure caps. (a) Narrow bore NMR compatible 4-port basic pressure caps (BC) for 5 and 10 mm OD NMR tubes. When sufficiently short tubes are used, an additional spinner is not necessary, as it is integrated into the pressure cap, resulting in a spinnerless cap (SC). (b) 5-port basic pressure caps (BC) compatible with 10 mm and 16 mm OD tubes, featuring one extra-large port (through-hole diameter 4 mm) designed for applications such as solvent evaporation via vacuuming. The smaller 4 ports were designed for 1/16″ or 1/32″ tubing.

While the aim was to increase the hyperpolarized contrast agent volume by using 16 mm OD tubes, our designs also included 5 and 10 mm versions for comparison. The smaller tubes are compatible with widely used commercial NMR tubes and high-resolution NMR systems. All reactor designs with 4 ports (Figure 5a) were compatible with narrow bore (NB) NMR devices: the “spinnerless cap” can be used by itself; basic caps should be used with adequately long tubes and corresponding alignment spinners.

The 4-port pressure caps were designed to allow for the injection of the precursor, infusion of purification components, pH_2_ bubbling, and exhaust. Due to the physical constraints of the NB NMR devices and the limited amount of precursor in these reactors, it was not possible to implement a large vacuum port in these designs. Alternatively, the exhaust port can be used as a vacuum port for solvent evaporation.

The fifth port on large designs was explicitly included to increase the evaporation rate of the solvent. By increasing the diameter of the evaporation port, more solvent in the gas form will be able to evaporate in a shorter time. This evaporation step is needed for the purification of the hyperpolarized solution if the evaporation of the solvent is chosen as the purification method.^27,28,43^ This expanded evaporation port is expected to shorten the time needed for this step.

While the glass tubes are cost-effective and commercially available, the property of the glass material makes them unusable under high pressure. To accommodate the weak mechanical properties of the glass material, the thickness of the walls must be increased, which, in turn, reduces the sample volume. Polymer tubes with a wall thickness of 1 mm, as opposed to 1.75 mm for glass tubes, offer a larger sample volume of ∼12%. Also, the 10 mm OD standard glass tubes cannot be used under pressures higher than 15 bar. In this case, polymer tubes are the only option for high-pressure hydrogenation, except sapphire tubes, which are expensive (10 mm OD, 7 mm ID, 7″ length, ∼750€/piece). Although polymers are not as chemically resistant as glass to solvents such as acetone, polymer tubes are the only cost-effective solution.

The designs were created and simulated with Autodesk Inventor Professional 2024 (Autodesk Inc.). The pressure inside the reactors was applied as a pressure load onto the inner walls of the reactors and the pressure caps. According to our simulations, the maximum stress values under 100 bar of pressure for the PEEK, PSU, and PEI reactors were 81.13, 91.61, and 93.21 MPa, respectively. The maximum displacement values were 637.2, 280.7, and 323.5 μm for 5, 10, and 16 mm tubes, respectively. All displacement values are within the elastic region, indicating that the reactors should not sustain any permanent damage from the tested pressure of 100 bar.

Out of 30 stress simulations under 100 bar of pressure load that were analyzed, only three simulations with PSU material resulted in values higher than the average yield tensile strength of the material,^44^ which are shown in bold in Table 2. As the PSU material has a lower mechanical stress value than PEEK and PEI and does not provide any extra advantages, the reactors were produced from PEEK and PEI materials.

**Table 2:** Stress Parameters of the Designed pH_2_ Reactors and Pressure Caps^a^

			max von Mises stress (MPa)			displacement (μm)		
	tube OD	element	PEEK	PSU	PEI	PEEK	PSU	PEI
4 ports	5 mm	BC	36.50	43.7	38.85	92.7	25.1	31.9
		SC	36.02	46.21	40.00	134.2	37.2	49.2
		PT	52.06	52.34	52.16	428.0	159.3	155.7
	10 mm	BC	63.80	58.37	59.18	63.8	15.6	20.7
		SC	81.06	**91.61**	82.57	283.7	64.5	87.8
		PT	47.86	47.91	47.88	535.8	178.1	183.6
5 ports	10 mm	BC	68.57	**89.45**	80.54	364.3	119.5	154.2
		PT	77.38	**89.30**	80.08	637.2	280.7	323.5
	16 mm	BC	39.63	64.83	51.07	118.5	51.2	60.9
		PT	81.13	82.22	93.21	571.1	209.6	199.6
max. average yield values from literature^44^			97.10	84.80	114.00	6585	3520	8075

a Maximum von Mises stress (MPa) and displacement (μm) values of the simulated reactors when a 100 bar pressure is applied (BC: basic cap, SC: spinnerless cap, PT: polymer tube; see Figure 5). The average tensile strengths for PEEK (97.10 MPa) and PEI (114.00 MPa) were higher than simulated values. Still, for PSU (84.80 MPa), on some setups, the simulated values were higher than average yield tensile strengths (shown in bold), meaning that the structure was likely to fail. No simulated displacement value exceeded the elongation at yield, indicating that the elasticity of the structures is protected.

### Injection Reproducibility

Before moving to hyperpolarization, we measured the reproducibility of the precursor injection into the reaction vessel with designed pressure caps. Only three ports of the pressure cap were required: (1) liquid injection, (2) pH_2_ pressure supply or liquid ejection, and (3) gas exhaust or N_2_ supply. Automatic valve control enabled reliable switching between these functions. Liquids were injected using a syringe pump (LA-110, Landgraf Laborsysteme HLL GmbH) equipped with a 20 mL syringe. The syringe was connected via a Luer lock adapter (IDEX, P-658) to 1/16 in PTFE tubing, which was inserted through one of the pressure cap ports and positioned approximately 5 cm from the bottom of the tube. For ejection, as in previous setups, N_2_ pressure was supplied, driving the liquid out through 1/16″ PEEK tubing (IDEX, 1538L). A 4-way valve, controlled by a servo motor, switched the function of this multipurpose tubing between pH_2_ supply and liquid ejection. In this manner, the entire process from injection to ejection was automated as previously described (see Figure 8c of ref 39).

In this experiment, 5, 10, and 16 mm glass tubes were filled to a height of 20 mm with acetone, and subsequently, the signal with a 90° pulse was measured. After that, the sample was ejected by pressurizing the tube and leaving one outlet open, through which liquids were ejected. This procedure was repeated automatically 10 times, and the entire experiment was repeated 3 times for each tube size (Figure 6a—shuttle). The coefficients of variation for each tube in the case of the injection series were 6.71%, 1.41%, and 0.63% for 5, 10, and 16 mm tubes, respectively.

**Figure 6:**
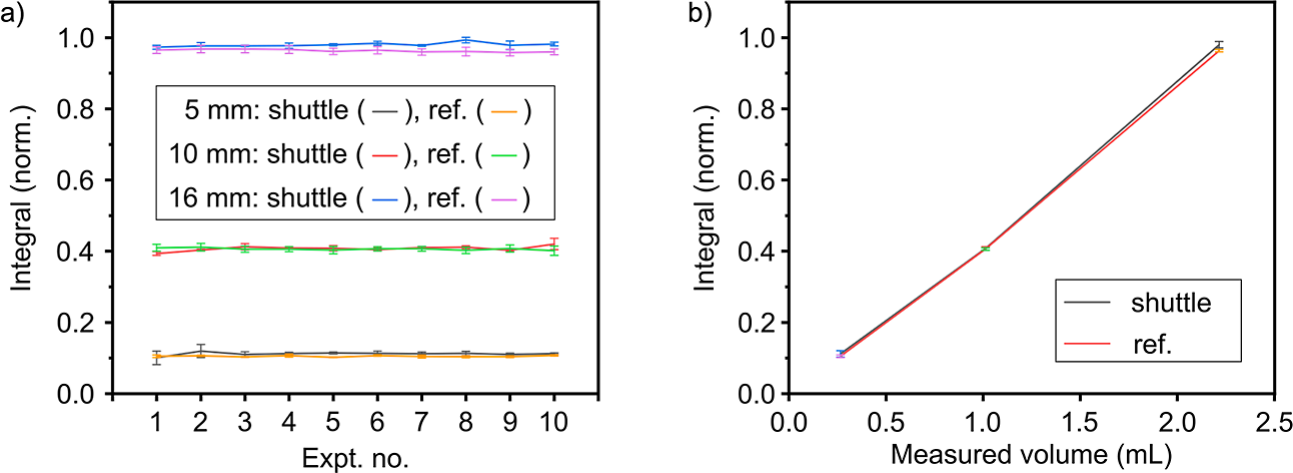
Sample injection reproducibility. (a) Reproducibility of signal integrals from 90° pulse experiments for reference (ref, manually pipetted volume) and automatically injected and ejected (shuttle) samples for 5, 10, and 16 mm tubes. Measurements show minimal variation across repetitions (with and without liquid shuttling). (b) The average integral of the spectra and standard deviation of all 30 spectra for each tube were plotted against the sample volume calculated from the measured sample weight. A correlation of 0.99994 was obtained.

The values of these integrals were also compared with the weighed amount of acetone (Figure 6b). The reference series was marginally more stable than the injection series, suggesting a slight increase in variability. Specifically, for the reference series, the coefficient of variation values were 2.50%, 2.23%, and 1.04% for 5, 10, and 16 mm tubes, respectively.

A high correlation between the obtained signal and the measured volume was observed. The measured volume (calculated from the weight) for 5 mm, 10 mm, and 16 mm tubes corresponded to the sample volumes of (0.265 ± 0.007), (1.013 ± 0.023), and (2.215 ± 0.023) mL, respectively.

Since the magnet exhibited a significant thermal drift of 1–2 ppm/mK, the resonance frequency adjustment was introduced right before excitation and measurement of the spectrum, which significantly increased long-term reproducibility. To assess the stability of the system, we repeated the experiment with manual pipetting of acetone into the same tubes and then measured the sample 10 times. This process was repeated three times for each tube (Figure 6—ref.).

### Optimizing ^1^H Sequence

As a model reaction, we chose the hydrogenation of vinyl acetate, which yielded ethyl acetate (EA) as the product. Fully protonated (VA-*h*_6_) and deuterated (VA-*d*_6_) precursors were compared.

An out-of-phase echo sequence with three composite refocusing pulses (45_X_-[τ-90_X_180_Y_90_X_-τ]_*n*=3_-FID) was used (Figure 7a). This sequence converted the antiphase PASADENA spectrum into an in-phase one, which was especially useful for inhomogeneous magnetic fields.^39,45–50^ All experiments presented here and in the following sections were performed by using a 16 mm tube and a pressure cap reactor made of PEEK material.

**Figure 7:**
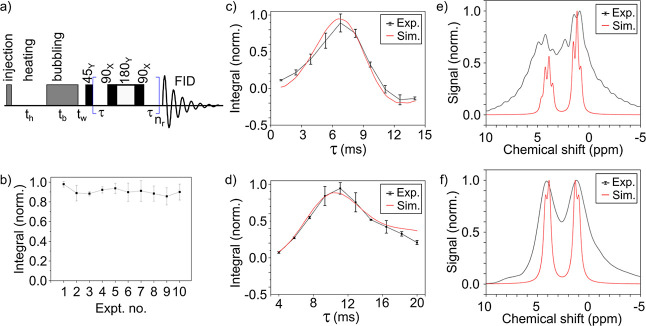
PHIP of VA with an out-of-phase echo sequence. Experiment scheme (a), hyperpolarization repeatability (b), and integrals of sequence (τ) dependences for VA-*h*_6_ (c) and VA-*d*_6_ (d), and corresponding spectra (e,f). (a) The experiment started with the injection of the precursor, a waiting period for heating of the sample, *t*_h_, the hydrogenation (bubbling) of the sample during the bubbling time with 52% enriched pH_2_, *t*_b_, a waiting period for the disturbed solution to settle down, *t*_w_, application of the OPE sequence with *n*_r_ = 3 refocusing and acquisition of the signal. (b) The reproducibility of the hyperpolarization process was measured by conducting two series of 10 experiments each, consisting of injecting, heating, bubbling, excitation, FID, and ejecting. The resulting standard variance for the obtained integrals was 6.79%. (c,d) Simulated (red lines) and experimental signal of EA-*h*_6_ (c) and EA-*d*_6_ (d), and corresponding spectra at optimum τ (e,f). The line width in simulated spectra was broadened homogeneously by 8 Hz, and 0 ppm corresponds to 18.717 MHz Larmor precession frequency. Simulations of OPE integrals (c,d) were obtained without considering relaxation or field inhomogeneity; obtained kinetics were multiplied by exp­(−τ/*T*_d_) with *T*_d_ = 60 ms (c) and 160 ms (d). Here, *t*_b_ was 10 s, *t*_h_ was 80 s, and *t*_w_ was 0.1 s, and the heater temperature was set to 40 °C.

Using the same injection protocol as that developed and described previously, we again performed three series of 10 experiments with 52% enriched pH_2_ for VA-*h*_6_ and VA-*d*_6_, where τ varied (Figure 7c,d). The optimal τ of 6.9 and 10.6 ms for EA-*h*_6_ and EA-*d*_6_ were found, and the obtained kinetics were well fitted with the simulations.

Finally, using VA and optimized τ, the polarization experiment was again performed in 2 series, 10 experiments in each: the resulting relative coefficient of variation of the mean was 6.79% (Figure 7b).

### Temperature Calibration

To determine the actual temperature of the sample, a thermocouple was used in conjunction with a data logger (Voltcraft K204 Thermometer, Conrad Electronic SE), which was passed through an unused port and fixed with a fitting. Then the injection cycles were repeated 3 times for different temperature settings of the heater (Figure 8a). It is not possible to heat the sample higher than the boiling point of acetone (56.2 °C, Figure 8a, gray dashed line) when the exhaust of the system is open, i.e., at ambient pressure. When, however, the exhaust was closed (Figure 8b 80 °C*), the pressure in the reactor could go higher, which allowed the temperature in the reactor to reach up to 63 °C in 100 s.

**Figure 8:**
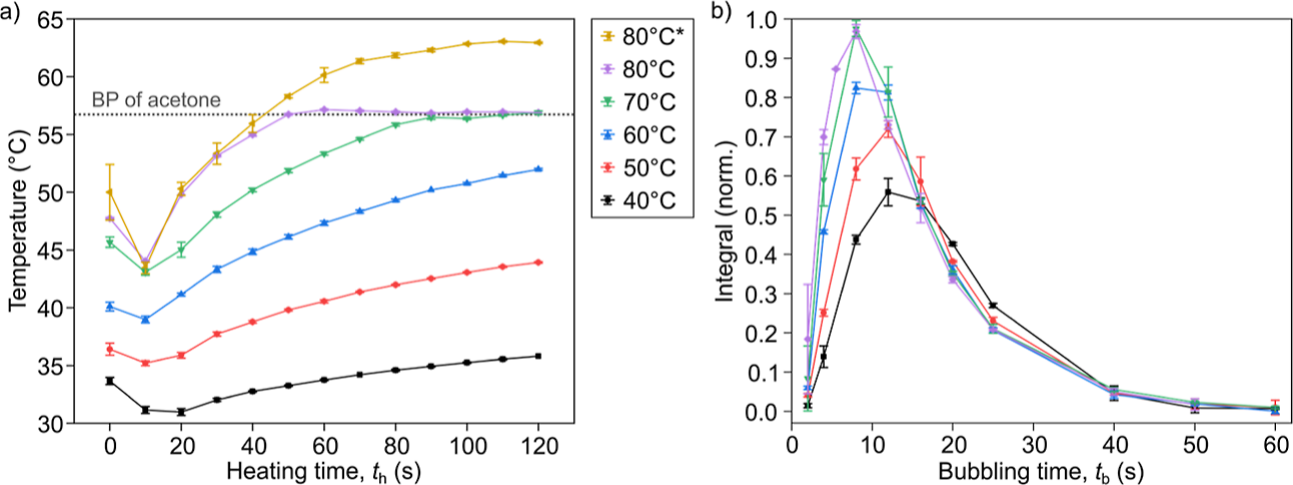
Temperature calibration and its effect on PHIP. (a) Temperature change recorded after injection of the 2.2 mL sample in a 16 mm OD tube under various heating temperature settings. The “80 °C*” temperature corresponds to the experiment when all exhausts of the reactor were closed, allowing pressure to build up inside the reactor, which enabled the acetone to reach higher temperatures. In all other cases, the sample was exposed to ambient pressure during heating. (b) Signal yield after hydrogenation of VA-*h*_6_ and application of optimized out-of-echo excitation after variable *t*_b_. The sample consisted of 50 mM VA, 5 mM Rh, and 2.2 mL of acetone. It can be observed that as the temperature increases, shorter bubbling times become more efficient.

It can be seen that the system did not reach the boiling point for any heater settings within 40 s; hence, all further experiments were conducted with this heating time (Figure 8a).

### Temperature Effect on PHIP

Hydrogenation times from 2 to 60 s were also investigated at varying temperatures (Figure 8b). With higher temperatures, it has been observed that shorter hydrogenation times are required to achieve the maximal signal.

### Pressure and pH_2_ Flow Effects on Reaction Kinetics

Then we assessed the effect of the pH_2_ pressure and flow on the PHIP signal (Figure 9). pH_2_ pressure had the advantage of increasing the amount of dissolved pH_2_ in the acetone. This had the potential advantage of increasing the interaction between pH_2_ and the catalyst, leading to accelerated hydrogenation. While the pH_2_ flow rate had a direct effect on the amount of delivered pH_2_ gas, it also affected the bubble size, hence contributing to the replenishment of pH_2_ in the solution.

**Figure 9:**
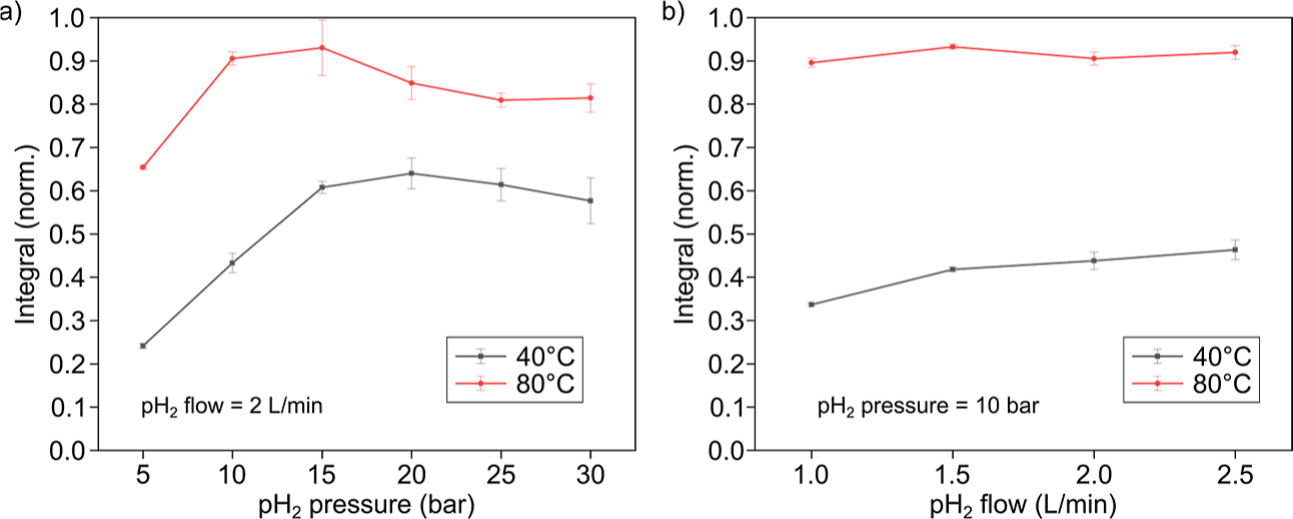
Effect of hydrogenation pressure (a) and pH_2_ flow rate (b). The bubbling time, *t*_b_, was 12 s for a 40 °C heating temperature; the temperature at the time of bubbling was 32.8 °C; 8 s for an 80 °C heating temperature; the temperature at the time of bubbling was 55.0 °C (see Figure 8b for optimum *t*_b_). For the hydrogenation pressure effect, the flow of pH_2_ was 2 L/min. For the pH_2_ flow rate effect, the hydrogenation pressure was 10 bar. For all experiments, the VA concentration was 50 mM, and the catalyst concentration was 5 mM.

It can be seen that the increased flow rate increased polarization only in the case of the 40 °C system: the signal grew by 37.7% when the flow changed from 1 to 2.5 L/min. With the 80 °C system, the flow rate did not have any obvious effect. The optimized parameters (92% enriched pH_2_, 20 bar pH_2_ pressure, 2 L/min flow, 4 s *t*_b_, 55 °C sample temperature for 2.2 mL of the sample in a 16 mm OD microwave tube, 50 mM VA conc., 5 mM Rh conc.) allowed us to reach ^1^H hyperpolarization of EA-*d*_6_ up to P = 31.3%.

## Discussion

In summary, the successful design and implementation of an automated, larger-volume hyperpolarizer capable of producing high polarization levels for biologically relevant substances while operating at high pressure were demonstrated.

The system’s unique integration of automated injection and gas handling, sample heating, high pressure and high volume availability, and rapid sample transfer allowed us to reach polarization levels of up to 31.3% for EA-*d*_6_ in acetone in a duty cycle of 80 s with an injection automation error of ∼14 μL (0.63%) and a coefficient of variation of polarization reproducibility of 6.79% from the mean.

The used pulse sequence yielded 50% ^1^H polarization in the ideal case and 100% of pH_2_, while 44.6% ^1^H polarization was obtained for 92% pH_2_, which was used. This demonstrates that we achieved 70% of the theoretical maximum ^1^H polarization (31.3/44.6 = 70%) while also increasing the polarization volume to 2.2 mL, which is sufficient for preclinical use. The achieved ^1^H molar polarization (product of concentration and polarization, see Section 2, Supporting Information)^51^ is calculated to be 31.3 mM for 2.215 mL of solution. If another sequence is used, which focuses polarization on one proton, then double polarization can be produced,^52^ which corresponds to 62.6% ^1^H in-phase polarization; however, the molar polarization will stay the same.

The *B*_0_ and *B*_1_ magnetic field homogeneity analysis results showed that although the FWHM of the 16 mm reactor was 2.492 ppm, the acquired images were of relatively good quality. Larger volumes of samples (*h* ≥ 30 mm) yielded noticeably worse image quality, as well as faster decaying nutation curves, compared to samples with *h* ≤ 20 mm, due to the poor homogeneity of *B*_0_ and *B*_1_.

The automation allowed us to optimize the following parameters reproducibly: sample temperature, pH_2_ pressure, pH_2_ flow, hydrogenation time, and sequence interpulse delays. The maximum was achieved at 15 bar of pH_2_, a flow of 1.5 L/min, an 80 °C heater temperature (or 55.0 °C sample temperature), and a hydrogenation time of 8 s. Since the increase of pH_2_ pressure and flow did not improve the polarization yield, we concluded that the 5 mM used catalyst is already saturated with the available amount of pH_2_. The estimated concentration of pH_2_ in the sample under 15 bar of hydrogenation pressure and 55 °C of temperature is ∼80 mmol/L.^53^ The decline in the polarization yield when the pressure exceeds 15 bar (Figure 9a) is tentatively attributed to vigorous bubbling and displacement of the sample mixture out of the volume of interest (VOI), resulting in less signal being acquired, and rapid hydrogenation compared to the used bubbling time.

Designed reactors with multiple connectors, including one large port for vacuuming, enabled robust injection, pressurization, evaporation, and purification of the samples under a relatively high pressure. The 16 mm microwave tube was found to be convenient as a reactor and a novel solution proposed here, as it provided excellent chemically stable properties and had a low cost of approximately 2€ per tube, which was similar to economy 5 mm NMR tubes but permitted experiments with pressures of up to 41 bar (600 psi) and at larger volumes.

Future studies will focus on automated, fast, and reliable purification of the hyperpolarized contrast agent following methodologies developed previously.^28,37,40,54^

## Methods

### Chemicals

As the catalyst of the hyperpolarization process, [1,4-bis­(diphenylphosphino)-butan]-(1,5-cyclooctadien)-rhodium­(I)-tetrafluoroborate ([Rh], 341134, Sigma-Aldrich) was used. Vinyl acetate (VA, 48486, Merck) and vinyl acetate-*d*_6_ (VA-*d*_6_, D-4097, CDN Isotopes) were used as precursors. Acetone (320110, Sigma-Aldrich) and acetone-*d*_6_ (444863, Sigma-Aldrich) were used as solvents for the preparation of the samples. The samples were prepared by mixing 50 mM of one of the two precursors (VA, VA-*d*_6_) with 5 mM [Rh] in acetone or acetone-*d*_6_. The prepared sample was loaded into a 20 mL syringe and used in the hyperpolarization experiment.

The pH_2_ that was used for hydrogenation was prepared using an in-lab-built pH_2_ generator that used liquid nitrogen as a coolant (52% enrichment)^20^ or with a two-stage cryosystem-based generator (92% enrichment).^22^

### Hydrogenation

#### Bubbling Time

The bubbling time is related to hydrogen saturation of the solution, which, in turn, affects the hyperpolarized precursor amount. Longer bubbling times usually mean a higher amount of precursor to be hydrogenated but also give more time for the hyperpolarized product to relax. In this study, 10 different bubbling times (2, 4, 8, 12, 16, 20, 25, 40, 50, and 60 s) were investigated. For 80 °C temperature experiments, the 5.5 s bubbling time was added and the 60 s bubbling time was omitted, while increased temperatures shortened the exchange time.

#### pH_2_ Pressure

Standard NMR tubes can be used up to 10 bar of pressure, but for higher pressures, a custom NMR tube out of PEEK and PEI with flanges must be produced. In order to find the optimal hydrogenation pressure, 5, 10, 15, 20, 25, and 30 bar of pH_2_ hydrogenation pressure values were tested.

#### pH_2_ Flow

The flow rates of 1.0, 1.5, 2.0, and 2.5 L/min were used. The flow of pH_2_ was measured with the flow controller (C100L, Sierra Instruments Inc.).

#### Sample Temperature

The temperature of the reaction has a shortening effect on the hydrogenation kinetics,^37^ which can be calculated as^39^

$$\left[PCH\right]=\frac{\left[{PC}_{0}\right]P_{0}}{1-R/k_{1}}\left(e^{-\tau_{b}R}-e^{\tau_{b}k_{1}}\right)$$

where [PCH] is the precursor hydrogenation concentration, [PC_0_] is the initial precursor concentration, *P*_0_ is the reached polarization, *R* is the reaction rate, *k*_1_ is the first-order rate constant of the reaction, and τ_b_ is the pH_2_ bubbling time. It is also proven that elevated temperatures have a positive effect on polarization levels.^30^

In order to elevate the temperature of the samples, a sample heating system (WTHA 1—Weller Tools GmbH) with hot air was implemented. The samples were heated to 40 °C, 50 °C, 60 °C, 70 °C, and finally 80 °C, which was the temperature limit of the sample heating system.

### Reactor Design, Simulation, and Production

#### General Description of the Hyperpolarization Reactor

We experimented with 4-port reactors, which can enable various liquid sample shuttling and delivery of pH_2_, and with 5-port reactors, where an additional large orifice tubing can be connected for rapid vacuuming of the solvent. All polymer parts were machined at the Central Workshop of the Biology Department and the Central Workshop of the Physics Department, CAU, according to the technical drawings (Supporting Information). The constituents of such reactors are described below.

#### Glass Reaction Vessel

Five mm (Boroeco-5–7, Deutero GmbH), 10 mm (Boroeco-10–7, Deutero GmbH), and 16 mm (908035, CEM GmbH) OD glass tubes were convenient vessels for hydrogenation experiments due to their chemical resistivity, low costs of ∼1–2€ per tube, and large opening (4.2 mm ID for 5 mm OD, 9.1 mm ID for 10 mm, and 12.5 mm ID for 16 mm OD) compared to high-pressure tubes; e.g., a high-pressure 10 mm NMR tube has an opening neck of 0.8 mm inner diameter (513-7PVH-7, Wilmad).

The designed pressure caps and reactors were suitable for standard 5 mm NMR tubes or 10 mm NMR tubes; the caps with integrated spinners were suitable for 4 in 5 mm NMR tubes (Boro-5-103.5-o, Deutero GmbH). 4 in 10 mm NMR tubes were uncommon, and most of the time, they had to be custom-cut by the supplier. The 16 mm microwave reactor tubes used with pressure caps were pressure-rated up to 600 Ψ (41 bar). The pressure caps can also be modified to suit, e.g., 35 mL microwave tubes with a 30 mm OD to enable even larger sample volumes (pressure-rated up to 20 bar, 24.5 mm ID, 909036, CEM GmbH).

#### 4-Port Pressure Caps

Two polymers, PEEK (polyetheretherketone) and PEI (poly­(ether imide)), were used for pressure caps and reactor vessels because of their high tensile strengths and chemical resistance to the solvents. Thirty mm diameter PEEK and PEI polymer rods (L. Buck & Sohn GmbH & Co.) were machined following the technical drawings (Supporting Information, Figures S2–S6). These pressure caps were equipped with four PEEK fittings and ferrules for 1/16″ tubing (F-333, F-142, IDEX Health & Science, LLC). Such a configuration was designed for 5 and 10 mm OD NMR tubes. The assembled pressure cells were compatible with the standard bore Bruker system. Optionally, one can use a PEEK sleeve (F-233, IDEX Health & Science, LLC) to decrease the diameter of the fitting tubes to 1/32”. Because of the integration of the spinner geometry into the pressure cap design, the use of a spinner can be eliminated with a 4 in-long glass NMR tube.

#### 5-Port Pressure Caps (4-Ports plus Vacuuming Port)

To enable rapid vacuuming, a larger port was necessary. This was designed only for 10 and 16 mm OD tubes. The current configuration of the pressure cap had a maximum diameter of 29.8 mm, which was incompatible with the standard bore Bruker system. However, it fits a wide-bore Bruker system and is compatible with our low-field MRI system. The vacuum port was introduced in the middle of the cells with a PEEK nut and ETFE ferrule (U-655, U-650, IDEX Health & Science, LLC, max 17 bar), enabling a connection to a vacuum pump through 1/4″ inch PA tubing (PA 1/4 SCHWARZ, Landefeld Druckluft and Hydraulik GmbH). When the system should withstand higher than 17 bar pressures, one can instead use a brass nut (GT 144 MS, Landefeld Druckluft and Hydraulik GmbH) to connect 1/4″ inch PA tubing to the vacuum port.

#### Polymer Reaction Vessel

To increase the hydrogenation pressure, polymer tubes are designed and produced from PEEK and PEI polymers. The simulations also include PSU (polysulfone) material, but the relatively weak material properties and low resistance to some solvents, e.g., acetone, precluded us from using it as a reaction vessel material. Five mm polymer reaction vessels are also not produced, due to their low volume and relatively hard machining processes originating from their small diameters and high tube lengths.

#### Sealing

To enable gas sealing of straight 5 and 10 mm OD NMR tubes, we used O-rings made from FFKM polymer for universal solvent resistance: 4.5 × 1.5 mm (1087924, Alwin Höfert KG) and 9.25 × 1.78 mm (1008515, NH O-RING GmbH & Co. KG), respectively. For the 16 mm OD tube with a flange, we used a PTFE (polytetrafluoroethylene) flat O-ring 13.5 × 2.25 mm dimensions with 2 mm height (DR 14 TE, Landefeld Druckluft and Hydraulik GmbH). Because of the geometry difference and the way the tubes were connected to the pressure cap, in the case of straight 5 and 10 mm flangeless NMR tubes, when the O-rings get wet, the tube can slide from the pressure cap. This was not the case for the 16 mm OD tube with the flange, in which case, only sealing can be compromised when the O-ring is wet.

#### Experimental Reactor Pressure Ranking

While the standard glass NMR tubes can withstand 10 bar of pressure (not specified by the manufacturer), the system with the 16 mm OD microwave tube can withstand at least 34 bar of pressure (specified by the manufacturer), which is the maximum pressure rating of the valves (P-782, IDEX Health & Science, LLC) used in the injection of liquids. The systems with 5 mm and 10 mm glass NMR tubes were pressurized up to 10.1 bar, and the system with the 16 mm microwave tube was pressurized up to 30.4 bar. No noticeable problem was seen.

#### Simulations of the Designed Reactors

The pressure caps and reactors were designed in Autodesk Inventor Professional 2024 (Autodesk Inc.), and the designs were loaded with 100 bar of pressure load on the related surfaces. Although this pressure value was much higher than the intended pressure value during experiments, the safety factor was kept higher to accommodate the errors of production, machining, material fatigue after pressurizing and depressurizing several cycles, and solvent degradation of the polymers, which could not be simulated in the finite element method (FEM) analysis. The mesh of the FEM analysis was configured to have a 5% average element size and a 2.5% minimum element size for the models.

#### Simulation of Spectra and OPE Performance

The expected spectra for EA-*h*_6_ and EA-*d*_6_ were simulated and compared with the experimental results (Figure 7e,f). The spectra were homogeneously broadened to imitate the experimental conditions. The performance of the OPE was also simulated and compared with experimental results. An exponential decay function, exp­(−τ/*T*_d_), was applied to the magnetization from the simulations to suppress oscillations.

#### Quantification of Hyperpolarization

The polarization level was calculated by using the reference thermal signal. For the thermal signal, the sample was measured 10 min after the hyperpolarization process, ensuring that all of the hyperpolarization signal had relaxed. After this measurement, the same sample was analyzed with a high-resolution, 9.4 T NMR system with a 5 mm broadband probe (400 MHz WB, NEO; BBFO, Bruker) to determine the amount of EA-*d*_6_ in the sample. The signal ratio of EA-*d*_6_ to the whole spectrum (Supporting Information, Figure S7) was used as a correction coefficient between the thermal and hyperpolarized signals measured in the polarizer in situ. This is necessary because portable MRI does not provide sufficient spectral resolution to assess the signal from EA-*d*_6_ exclusively.

## Supplementary Material



## Data Availability

All the raw data, technical drawings, and all used MATLAB scripts to simulate OPE kinetics, NMR spectra, and *B*_1_ field distribution are available on the Zenodo repository (https://doi.org./10.5281/zenodo.16984202).

## Notes

*B*_1_ field distribution simulations, technical drawings, and high-resolution ^1^H NMR spectra of the hydrogenated product (PDF)
