# Supplementary material for: Upscaling the Hyperpolarization Sample Volume of an Automated Hydrogenative Parahydrogen-Induced Polarizer
Source: ACS Meas Sci Au. 2025 Oct 6;5(6):857–67. doi: 10.1021/acsmeasuresciau.5c00097 (PMC12715744; doi:10.1021/acsmeasuresciau.5c00097)
Supplement: Supplementary file 1 [file tg5c00097_si_001.pdf]

Supplementary Information

Upscaling the hyperpolarization sample volume of an automated hydrogenative parahydrogen-induced polarizer

Yenal Gökpek[a], Jan-Bernd Hövener*[a], Andrey N. Pravdivtsev*[a]

[a] Section Biomedical Imaging, Molecular Imaging North Competence Center (MOIN CC), Department of Radiology and Neuroradiology, University Hospital Schleswig-Holstein, Kiel University, Am Botanischen Garten 14, 24114, Kiel, Germany

Contents

1. Simulations of nutation curves of the B_1 coil with different sample heights 2
2. Calculation of molar polarization...3
3. Technical drawing of the 5 mm spinnerless cap with polymer tube.......................................4
4. Technical drawing of the 5 mm basic cap...5
5. Technical drawing of the 10 mm spinnerless cap with polymer tube.....................................6
6. Technical drawing of the 10 mm basic cap..8
7. Technical drawing of the 16 mm basic cap..9
8. Spectra of the product with high-resolution NMR 11
9. Relaxation of the mispositioned samples..12

1. Simulations of nutation curves of the B_1 coil with different sample heights

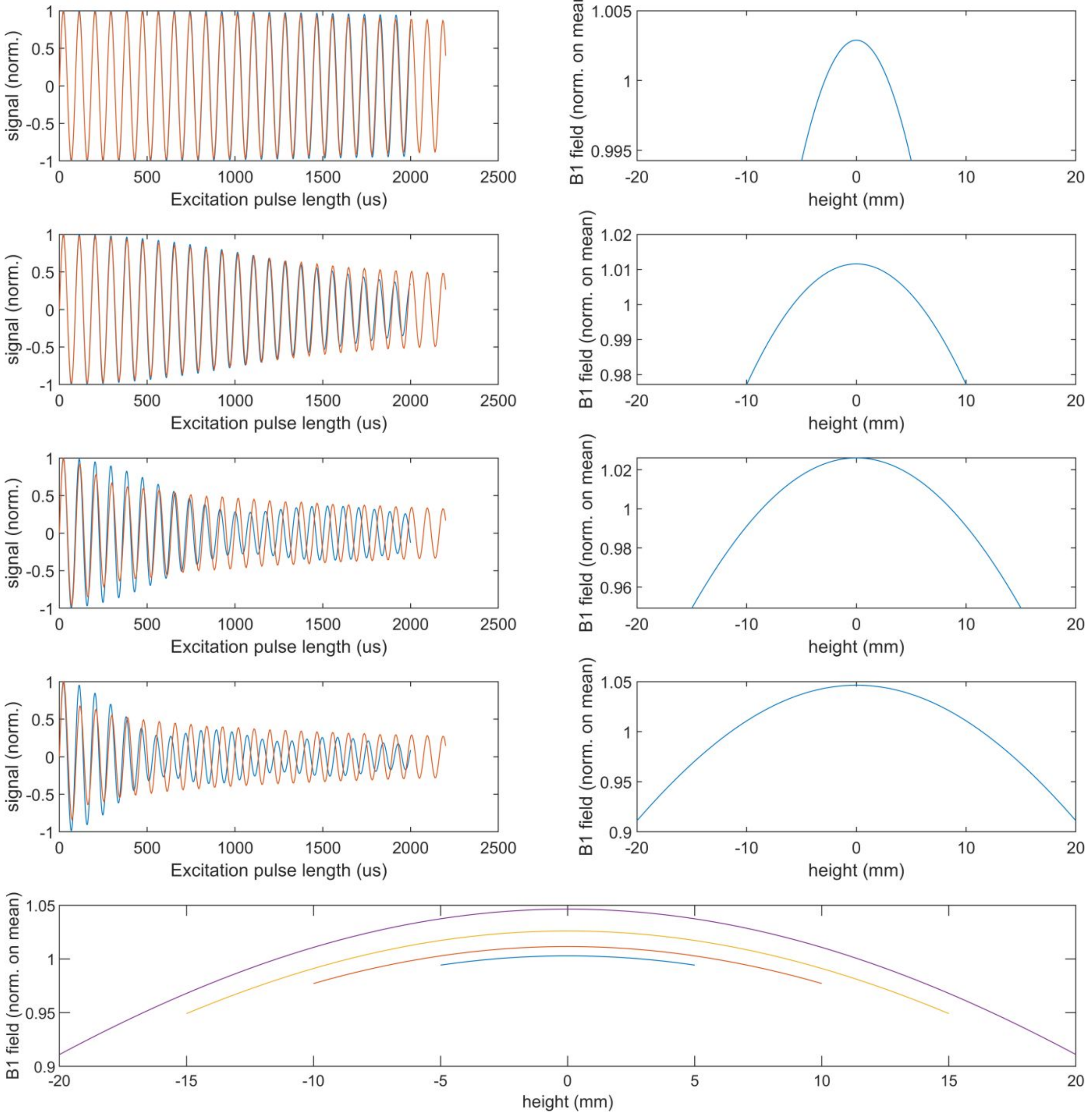

Figure S1. Simulated nutation decay parameters for 10, 20, 30, and 40 mm high acetone in a 16 mm reactor.

2. Calculation of molar polarization

Molar hyperpolarization is a way of expressing how much nuclear spin polarization is achieved per mole of substance. It is defined as:

$$P_{mol} = P \cdot c$$

where P is nuclear spin polarization (in absolute units, not in %), and *c* is the molar concentration (in mol/L) of the hyperpolarized substance. This value is also calculated because it indicates the number of polarized nuclear spins available per unit volume.

We measured the average ^{1}H polarization of 50 mM EA-d6 as 31.3%, using thermal polarization as a reference. Because we employed an out-of-phase sequence, the polarization is observed on two protons. This means our total molar polarization ($P_{\mathrm{mol,tot}}$) could be calculated as:

$$P_{\mathrm{mol,tot}} = P \cdot c \cdot n_{\mathrm{H}}$$

where n_{H} is the number of hyperpolarized protons. In our case, 2 protons (**Figure 1**, second molecule, red protons). This corresponds to the following molar polarization of the sample:

$$P_{\mathrm{mol,tot}} = 0.313 \times 50\ \mathrm{mM} \times 2 = 31.3\ \mathrm{mM}$$

3. Technical drawing of the 5 mm spinnerless cap with polymer tube

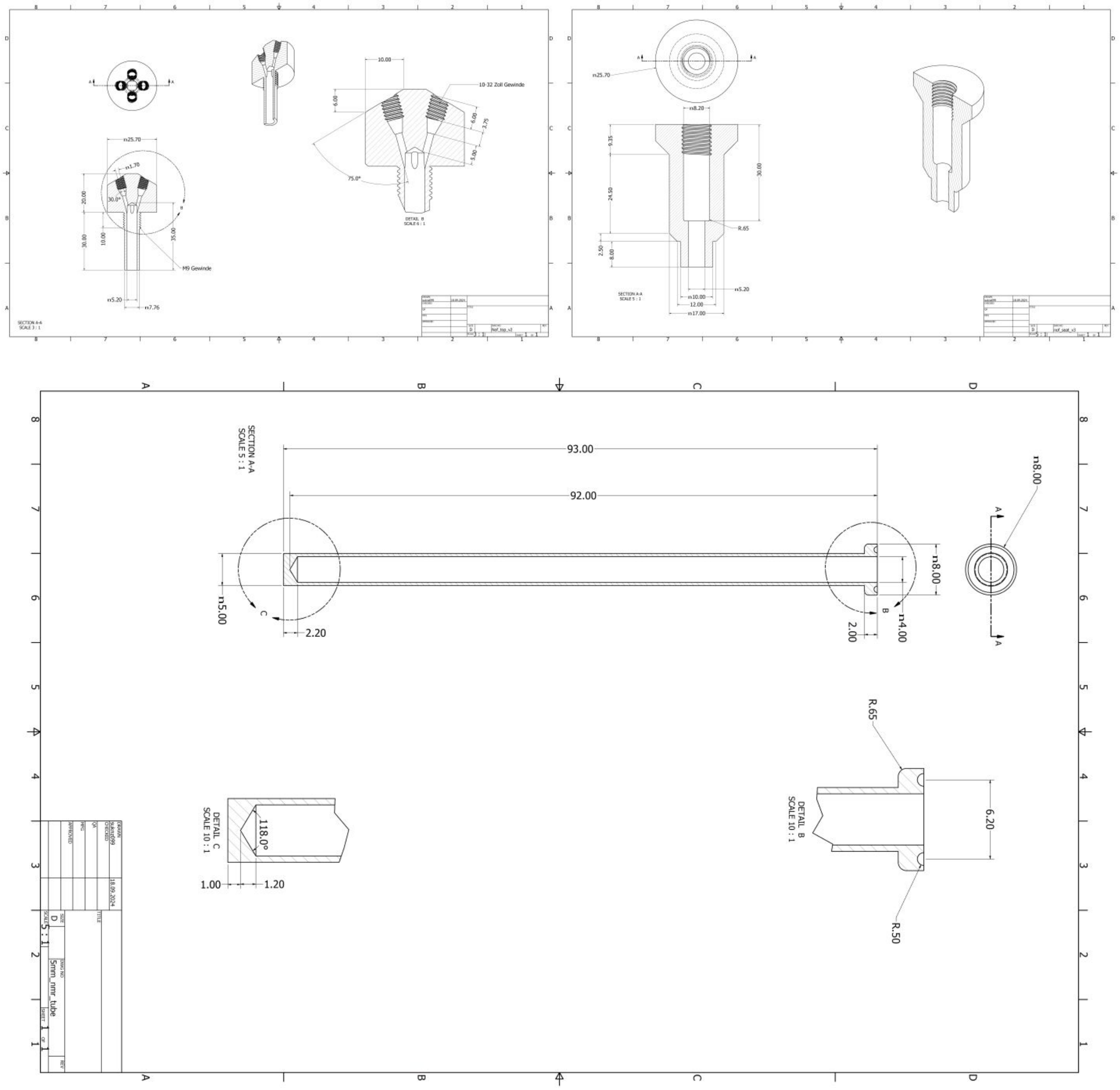

Figure S2. 5 mm spinnerless cap with polymer tube technical drawing

4. Technical drawing of the 5 mm basic cap

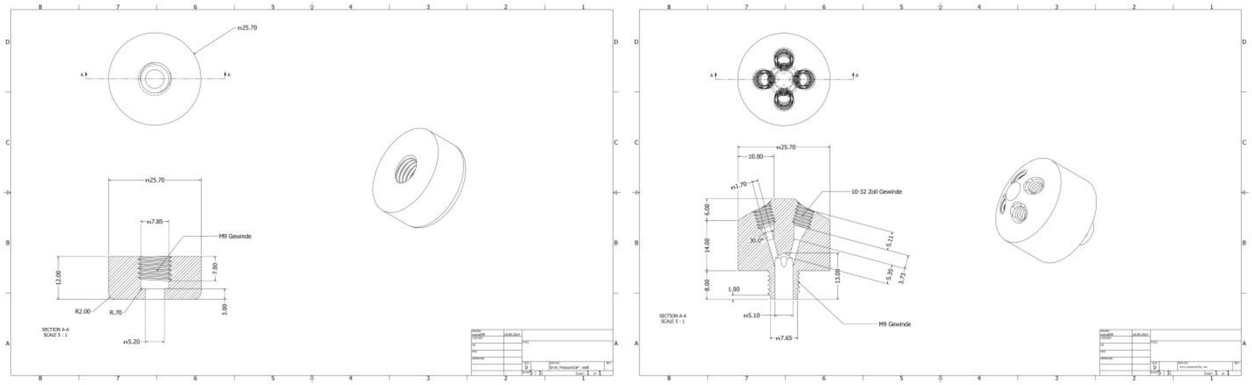

Figure S3. 5 mm basic cap technical drawing

5. Technical drawing of the 10 mm spinnerless cap with polymer tube

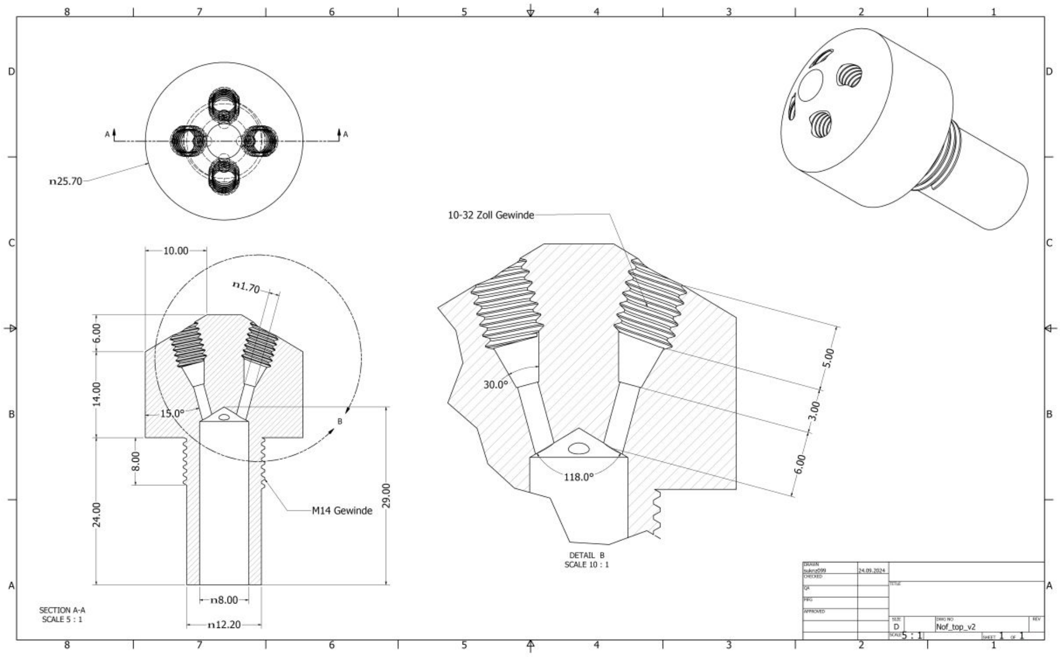

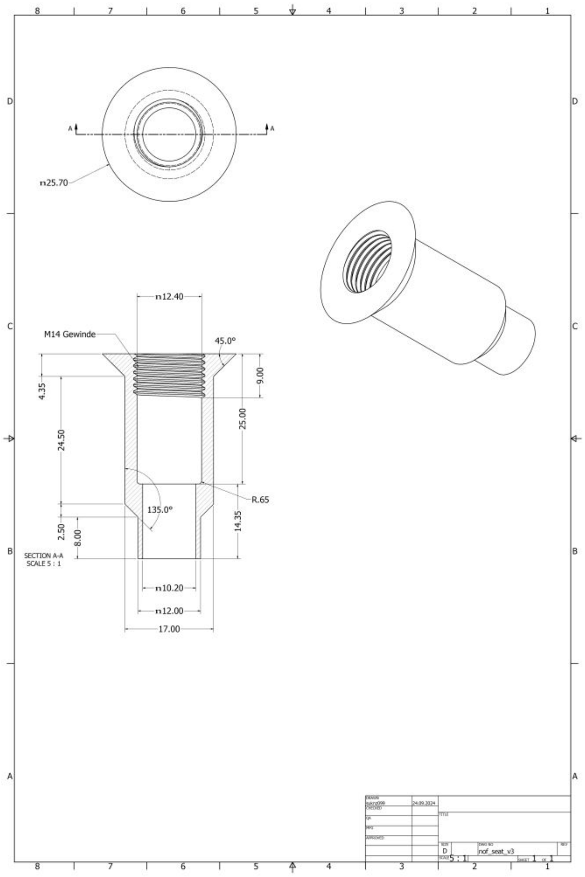

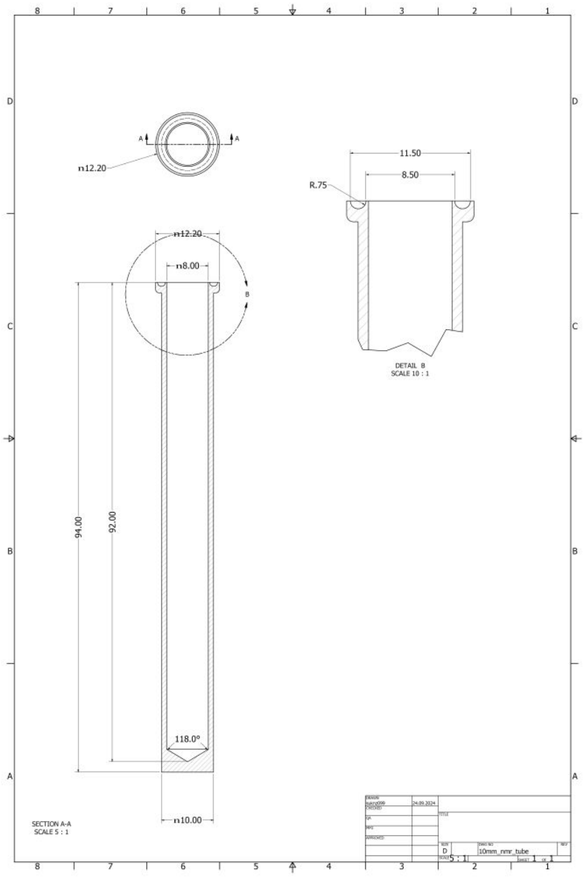

Figure S4. 10 mm spinnerless cap with polymer tube technical drawing

6. Technical drawing of the 10 mm basic cap

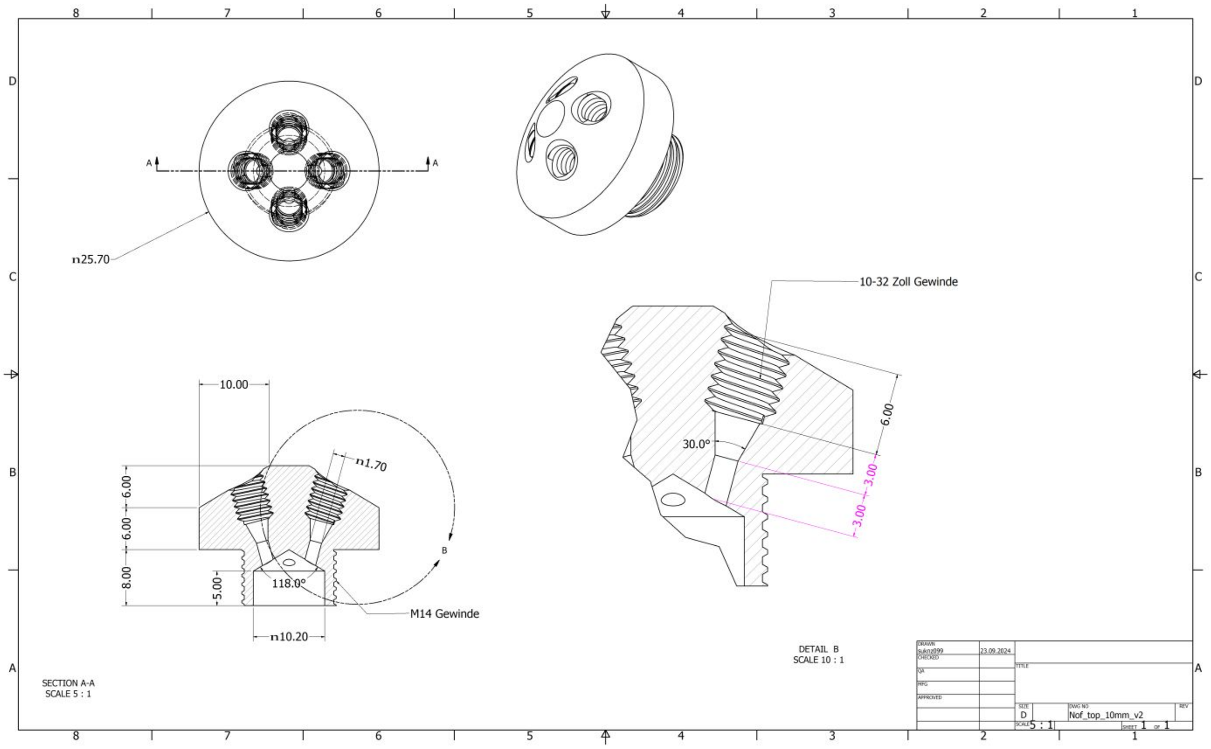

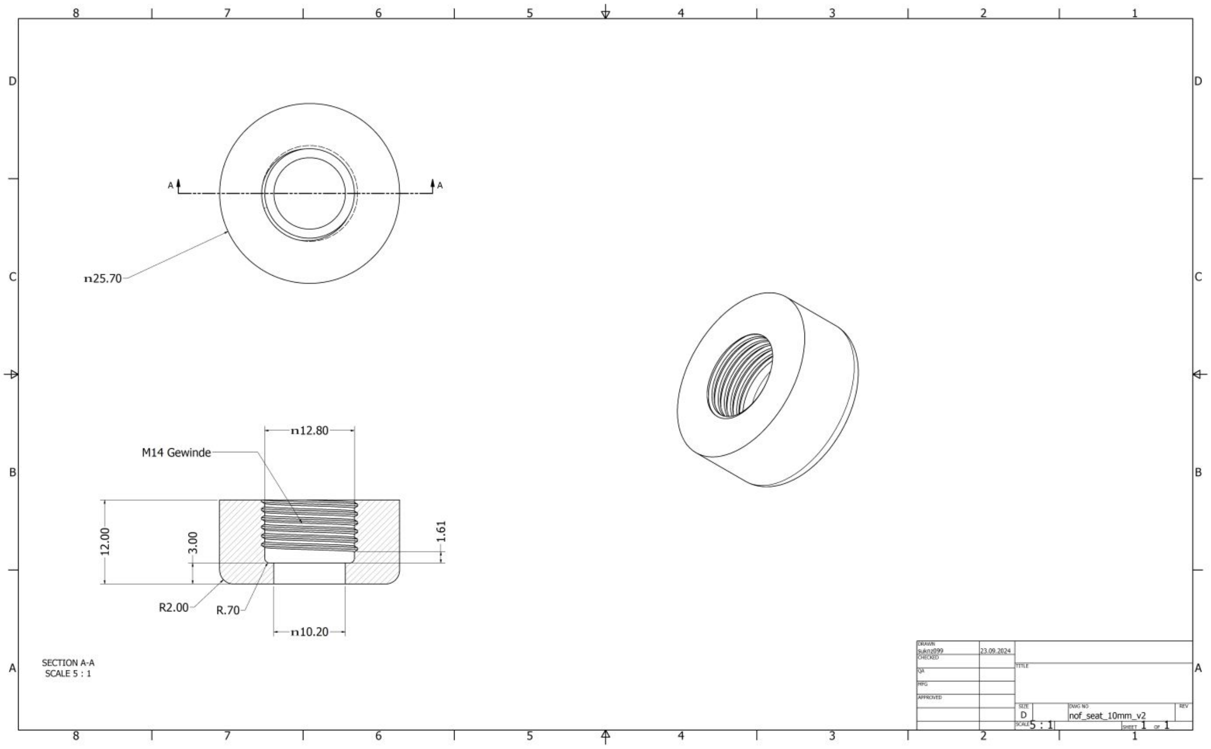

Figure S5. 10 mm basic cap technical drawing

7. Technical drawing of the 16 mm basic cap

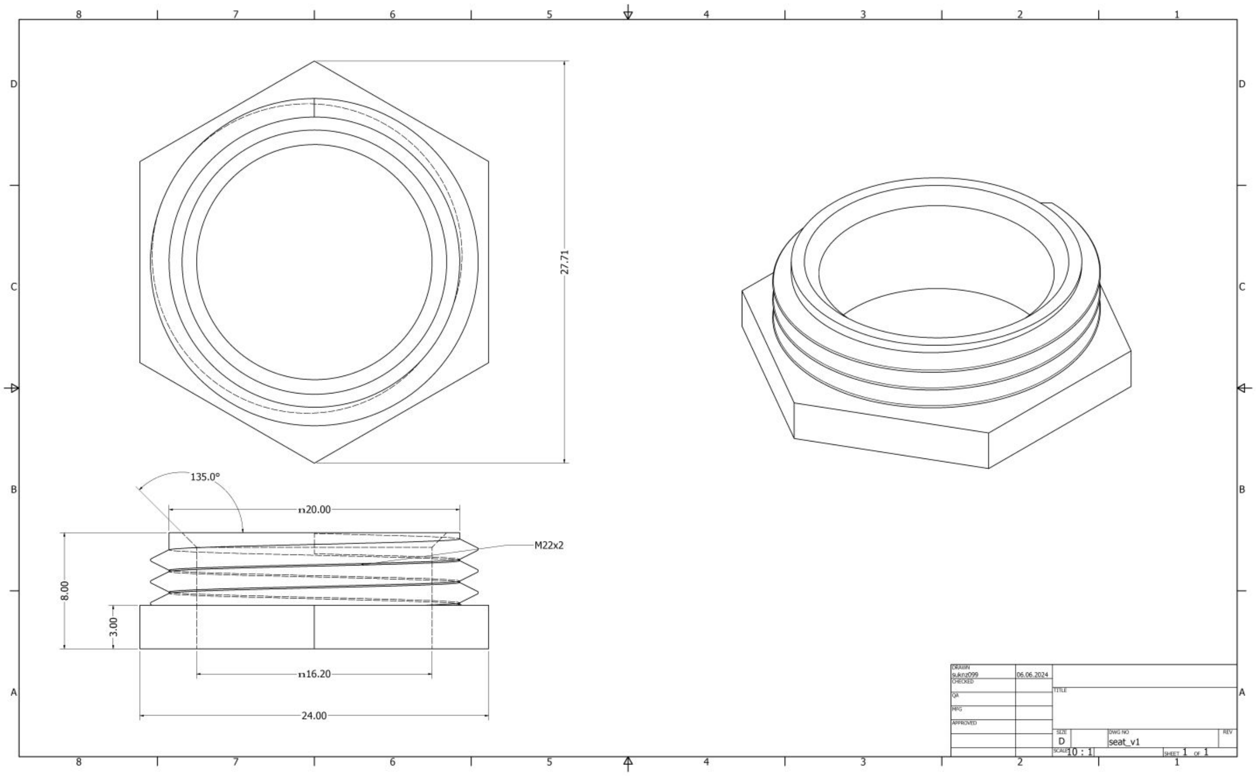

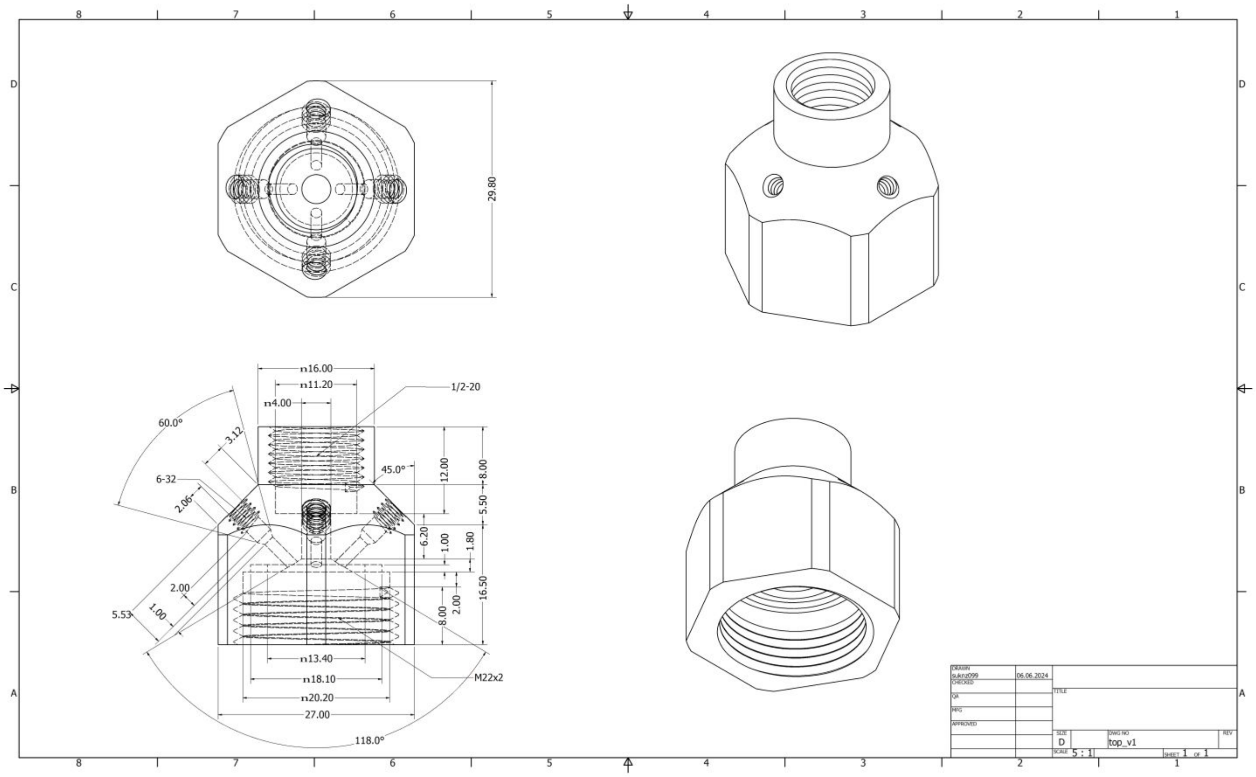

Figure S6. 16 mm basic cap technical drawing

8. Spectra of the product with high-resolution NMR

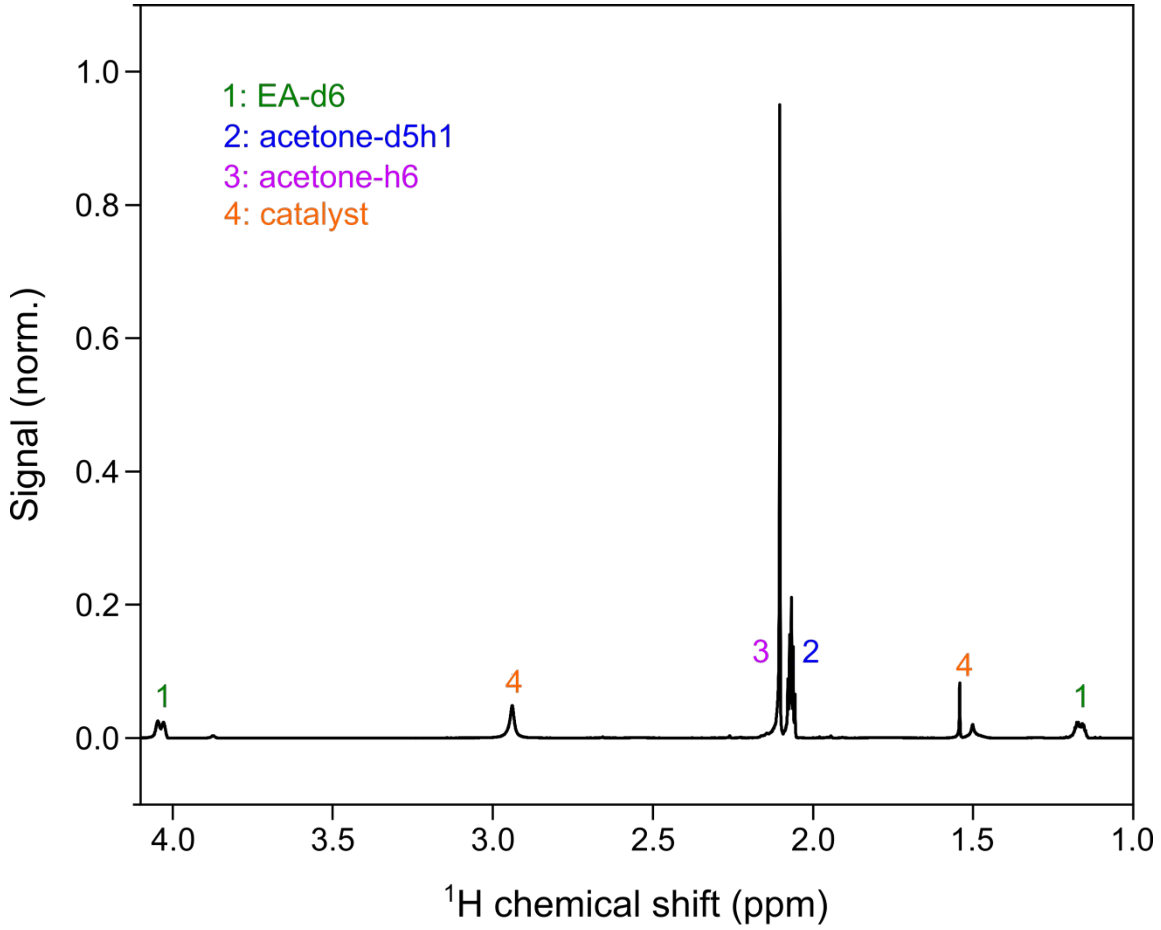

Figure S7. High resolution spectrum of the product

9. Relaxation of the mispositioned samples

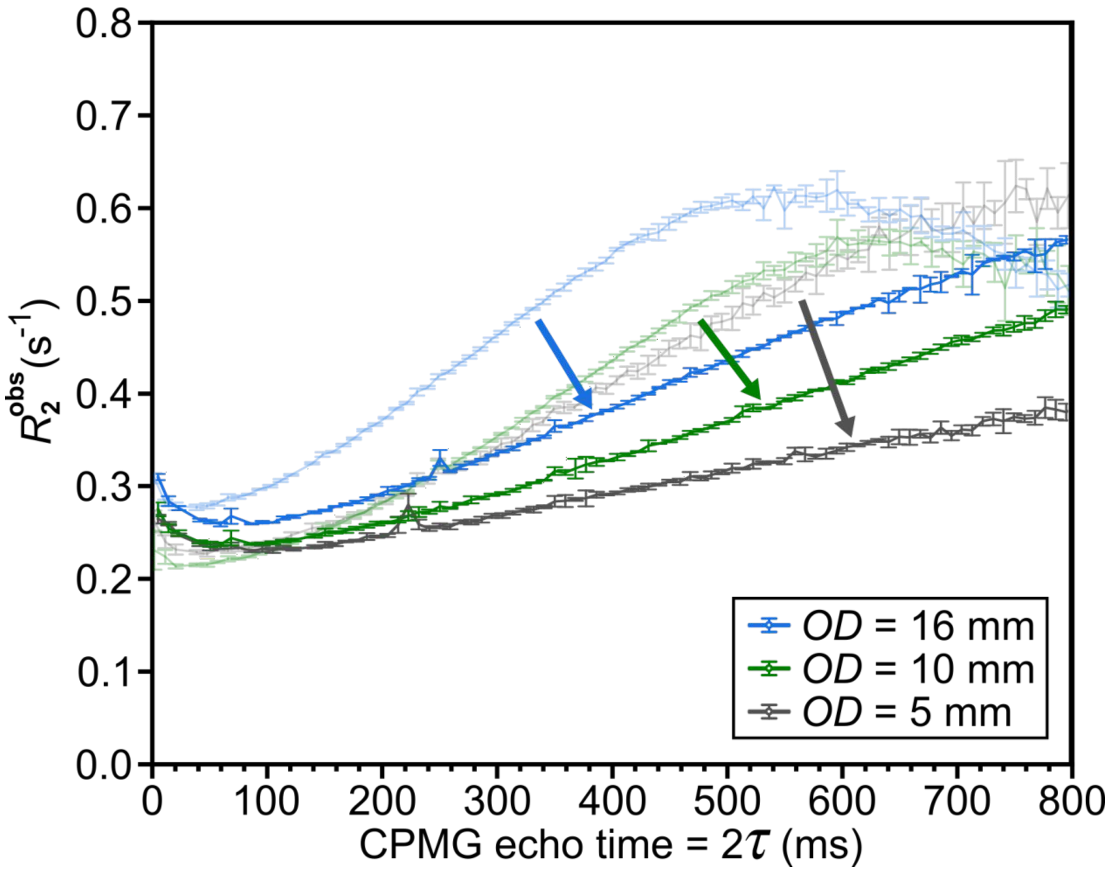

Figure S8. Relaxation of the mispositioned samples (shown in semi-transparent) caused by extra inhomogeneous B_0 and B_1 fields. Inhomogeneity could lead to the appearance of erroneous maxima in observed R_2.
